# Myocardial Calcium Handling in Type 2 Diabetes: A Novel Therapeutic Target

**DOI:** 10.3390/jcdd11010012

**Published:** 2023-12-30

**Authors:** Abhishek Dattani, Anvesha Singh, Gerry P. McCann, Gaurav S. Gulsin

**Affiliations:** Department of Cardiovascular Sciences, University of Leicester and NIHR Leicester Biomedical Research Centre, Leicester LE3 9QP, UK; as707@leicester.ac.uk (A.S.); gpm12@leicester.ac.uk (G.P.M.); gg149@leicester.ac.uk (G.S.G.)

**Keywords:** diabetes, calcium handling, manganese-enhanced MRI

## Abstract

Type 2 diabetes (T2D) is a multisystem disease with rapidly increasing global prevalence. Heart failure has emerged as a major complication of T2D. Dysregulated myocardial calcium handling is evident in the failing heart and this may be a key driver of cardiomyopathy in T2D, but until recently this has only been demonstrated in animal models. In this review, we describe the physiological concepts behind calcium handling within the cardiomyocyte and the application of novel imaging techniques for the quantification of myocardial calcium uptake. We take an in-depth look at the evidence for the impairment of calcium handling in T2D using pre-clinical models as well as in vivo studies, following which we discuss potential novel therapeutic approaches targeting dysregulated myocardial calcium handling in T2D.

## 1. Introduction

Heart failure (HF) has been acknowledged as a common, underappreciated complication of type 2 diabetes (T2D) [1], with T2D leading to a two- and five-fold increased risk of HF in males and females respectively [2,3,4]. Large-scale observational studies have shown that whilst other complications of T2D are mitigated by strict risk factor management, this does not hold true for the risk of HF [3].The diagnosis of HF portends a poor prognosis with a five-year mortality of 56% in a UK-based study [1].

Animal models, post-mortem and non-invasive imaging studies have shown that T2D is associated with numerous cardiac structural and functional perturbations, which occur independent of traditional risk factors and precede the development of overt HF [2,5,6,7,8,9,10,11]. These changes include left ventricular (LV) remodeling [6,12,13,14,15,16], diastolic and systolic dysfunction [12,13,17,18], coronary microvascular dysfunction [14], impaired myocardial energetics [19] and cardiac steatosis [20]. Whilst numerous mechanisms have been proposed to contribute to this distinct diabetic cardiomyopathy, a major yet overlooked contributor is abnormal myocardial calcium handling.

In this review, we describe the physiology of myocardial calcium handling and summarize emerging evidence for its central role in the pathogenesis of HF in T2D. We highlight the potential for novel imaging techniques to allow in vivo assessment of myocardial calcium uptake. Lastly, we describe the application of novel therapeutics targeting perturbed calcium handling in T2D.

## 2. Physiology of Myocardial Calcium Handling

The importance of calcium in myocardial function was first described in 1883 [21] and the concept of ‘calcium-induced calcium release’ within the myocardium was subsequently introduced in 1978 [22]. Since then, there have been significant advances in our understanding of the role of calcium as a key link in excitation-contraction coupling, a term that describes communication between electrical events and cardiac muscle contraction [23].

**Figure 1:**
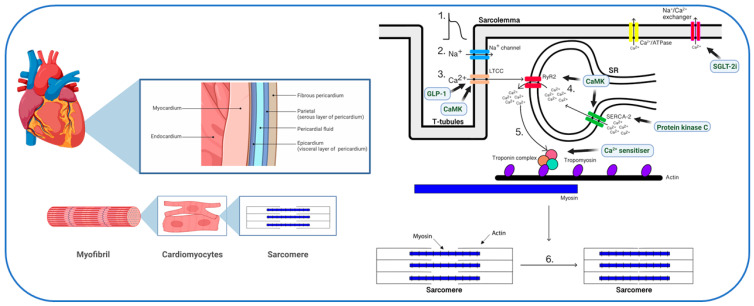
Myocardial calcium handling and potential therapeutic targets. **Left**: within the myocardium, the cardiomyocyte is made up of myofibrils that are formed of sarcomeres, which are the contractile units. **Right**: Steps in the excitation-contraction coupling pathway: 1. Arrival of action potential; 2. Influx of sodium; 3. Influx of calcium via the LTCC; 4. Calcium binds to RyR2 on the SR; 5. Release of calcium from SR; 6. Contraction of cardiomyocyte. Abbreviations: LTCC = L-type Ca^2+^ channels; SR = sarcoplasmic reticulum; RyR2 = ryanodine receptor; SERCA-2 = sarcoplasmic reticulum Ca^2+^ ATPase-2, GLP-1 = glucagon-like peptide-1 receptor agonist, SGLT-2i = sodium glucose co-transporter 2 inhibitor, CaMK = calmodulin dependent protein kinase II. Block arrows show potential novel therapeutic targets for myocardial Ca^2+^ handling in type 2 diabetes. Created with BioRender.com, accessed on 23 October 2023.

The contractile unit of the myocyte is the sarcomere, which is composed of myosin and actin filaments in addition to tropomyosin and the troponin complex (Figure 1) [24]. The myosin head contains the binding site for actin, which is required for cross-bridge formation and sarcomere shortening. Tropomyosin is attached to actin and prevents calcium binding. The troponin complex is formed of troponin T, I and C, and this is bound to tropomyosin. In the relaxed state, phosphorylated troponin I reduces the binding of calcium to troponin C. It is, however, the binding of calcium that leads to a conformational change resulting in cross-bridge formation between filaments, thus producing the pressure development that is required for ejection of blood from the ventricle [24,25].

Excitation-contraction coupling (Figure 1) begins with the arrival of an action potential at the cardiac myocyte. As a result of depolarization, initially there is a rapid influx of sodium ions via the voltage-gated sodium channels which are located at the cell membrane, known as the sarcolemma [26]. This is followed by the activation of voltage-gated L-type calcium channels (LTCC), also within the sarcolemma, resulting in a small but crucial influx of calcium ions. This influx results in calcium binding to ryanodine receptor-2 (RyR2) on the sarcoplasmic reticulum (SR), which in turn leads to a large and fast release of calcium from the SR into the cytosol [24]. Although the LTCC directly only leads to a small influx of calcium, the overall impact it has is a 100-fold increase in cytosolic calcium concentration, mainly released from the sarcoplasmic reticulum.

The subsequent extensive release of calcium allows the binding of calcium to troponin C, leading to destabilization of the troponin-tropomyosin complex from the actin-myosin binding site. This results in cross-bridge formation and allows myosin to pull the actin filament producing tension. Subsequent hydrolysis of adenosine triphosphate (ATP) and the binding of further ATP allows the release of the cross-bridge and further cross-bridges to form [24,26]. Relaxation of the sarcomere is an active, energy-requiring step utilizing ATP and SR calcium ATPase (SERCA-2). This channel allows the reuptake of calcium back into the SR, but its activity is facilitated by the phosphorylation of phospholamban and by direct phosphorylation by calmodulin-dependent protein kinase II [27].

Additional mechanisms of calcium release from the cytosol include the calcium/ATPase and sodium/calcium exchangers, which are both located at the sarcolemma. The sodium/calcium exchanger is also able to reverse its direction of transport at the peak of action potential, resulting in an influx of calcium [28]. Furthermore, there are calcium binding proteins within the cytosol such as calmodulin and calsequestrin. Calmodulin can bind to calcium to form a complex that can activate the calcium/ATPase within the sarcolemma and thus further increase the release of calcium. Calsequestrin allows the storage of calcium and also plays a role in regulating RyR2 [24,26,29]. Mitochondrial storage may also play a role in calcium homeostasis within the cardiac myocyte [30]. In summary, calcium release and reuptake are essential for effective systolic and diastolic function of the heart [15,31].

## 3. Calcium Handling in Heart Failure

**Figure 2:**
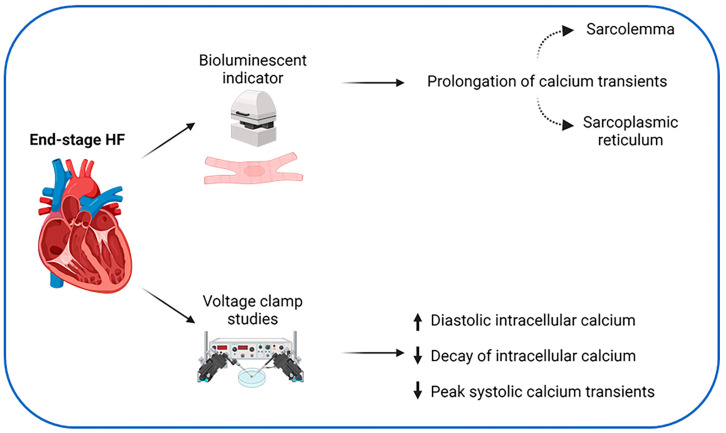
Initial experiments showing abnormalities in calcium handling in the failing human heart. Biopsy samples from patients with end-stage HF showed various abnormalities of myocardial calcium handling. Created with BioRender.com, accessed on 23 October 2023.

Heart failure is a common clinical syndrome that can be classified into three main categories: heart failure with reduced ejection fraction (HFrEF), mildly reduced ejection fraction (HFmrEF) or preserved ejection fraction (HFpEF) [32]. Altered calcium handling in the failing human heart (Figure 2) has been recognized since 1987 [33], with a comparison of explanted hearts from patients with end-stage HFrEF undergoing transplantation to those from organ donors who died of non-cardiac causes. With the use of a bioluminescent indicator for calcium called aequorin, measurement of intracellular calcium transients is possible. When stimulated to contract, the cells from papillary muscles of patients with HF demonstrated prolongation of tension development and marked delay in relaxation. This was associated with a prolongation of calcium transients and the duration of the action potential. They also found that changes in calcium transients consisted of two components and these were a result of abnormal calcium handling at the level of the sarcolemma and SR. Of note, the end-stage HF cohort in this study included a mixture of disease such as ischemic, non-ischemic and hypertrophic cardiomyopathy suggesting that calcium dysregulation may be a final common pathway.

Further work showed that in patients with dilated cardiomyopathy, dihydropyridine binding sites, which represent the number of calcium channels, were not reduced [34], implying that changes in influx of calcium could rather be related to dysfunctional channels. Voltage clamp studies using fluorescent indicators in cardiac myocytes from patients with end-stage HF have shown an increase in diastolic intracellular calcium, a slower rate of decay of intracellular calcium due to reduced reuptake of calcium at the SR, as well as smaller peak systolic calcium transients [35]. Other investigators have confirmed these results but did not show difference in diastolic intracellular calcium, cellular calcium buffering or in the LTCC current [36]. In the failing hearts of patients with dilated and ischemic cardiomyopathy, defects in dephosphorylation can also lead to an increased availability of open LTCC [37]. This can result in further efflux of calcium via the RyR2 at the SR [38] and an overall result of cytosolic calcium overload. A reduction in SERCA-2 expression and activity further explains the increased end-diastolic cytosolic calcium often seen in heart failure [39,40]. Mitochondrial calcium concentration is reduced in HFrEF which leads to increased reactive oxygen species, impaired myocardial energetics and impaired contractility [41].

In HFpEF, distinct changes have also been noted. Left ventricular biopsies from patients with HFpEF have demonstrated increased T-tubule density [42] and animal models of HFpEF have shown increased calcium transients [43]. As opposed to in HFrEF, there is an increase in mitochondrial calcium concentration [44]. Together these features may help compensate for the increased myocardial stiffness and prevent an impairment in systolic function in HFpEF [45].

It is clear that there are changes in calcium homeostasis within cardiac myocytes in patients with end-stage HF secondary to ischemic, dilated and hypertrophic cardiomyopathy, but these studies did not specifically assess diabetic cardiomyopathy. They also did not investigate patients with HF with preserved ejection fraction, in whom about 45% are known to have T2D [46], and they did not specifically assess the impact of T2D on the mechanisms described.

## 4. Calcium Handling in Diabetic Cardiomyopathy

### 4.1. Animal Studies

**Figure 3:**
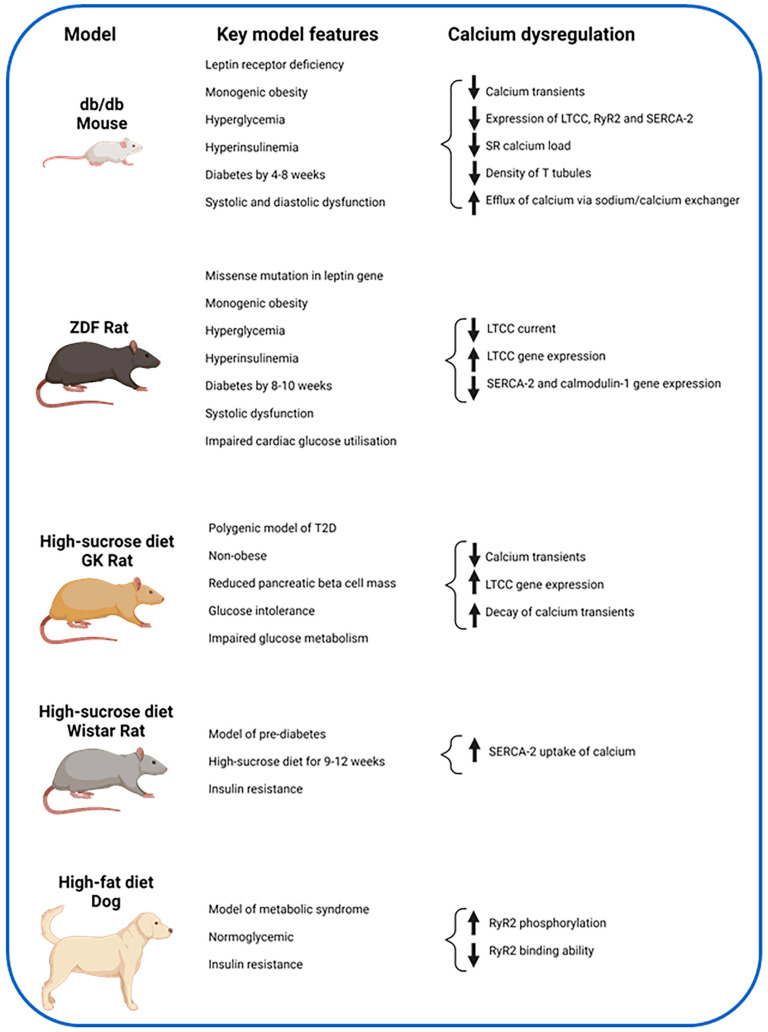
Key features of abnormal myocardial calcium handling demonstrated by pre-clinical models. Abbreviations: ZDF = Zucker Diabetic Fatty, GK = Goto-Kakizaki, LTCC = voltage-gated L-type calcium channels, RyR2 = ryanodine receptor-2, SR = sarcoplasmic reticulum, SERCA-2 = SR calcium adenosine triphosphatase. Created with BioRender.com, accessed on 23 October 2023.

Several animal models (Figure 3) [47,48,49,50] have been used to investigate alterations in myocardial calcium handling in diabetic cardiomyopathy (Table 1). These have primarily been limited to murine and canine models, and have focused on LTCC, RyR2 and SERCA-2.

#### 4.1.1. LTCC

Using two-photon and confocal microscopy, db/db mice with impaired LV fractional shortening have been found to have decreased intracellular calcium transients within the whole heart and on a cellular level [53]. Furthermore, western blots showed that these mice had a 38% decrease in the expression of LTCC which was correlated significantly with the 31% decrease in macroscopic calcium current they observed. Interestingly, they found that the functionality of individual LTCC in these mice were normal, which is contrary to the post-mortem human studies described above suggesting differences in the mechanisms of calcium dysregulation present in end-stage HF and diabetic cardiomyopathy. These findings were accompanied by a reduction in SR calcium load, reduced expression of RyR2 and increased efflux of calcium via the sodium/calcium exchanger. Other investigators have verified these results [54] but have also shown reduced density of T tubules in sedentary diabetic mice and reduced SERCA-2 expression.

Supporting evidence for the central role of calcium mishandling in diabetic cardiomyopathy has been demonstrated using the ZDF rat, in which ventricular myocytes showed impaired shortening and time to peak shortening [55]. These myocytes have an upregulation of the expression of genes which encode subunits on LTCC including *Cacna1c* (LTCC alpha 1C subunit), *Cacna1g* (LTCC alpha1G subunit) and *Cacna2d1* (LTCC alpha2/delta subunit) when compared to age-matched controls. They also have downregulation of the expression of the genes *Atp2a2* and *Calm1* which encode SERCA-2 and Calmodulin-1, respectively. The authors also found a reduced LTCC current. Together, these findings may suggest upregulation of gene expression for LTCC subunits and downregulation of those encoding SERCA-2/Calmodulin-1 may represent a compensatory mechanism to maintain cytosolic calcium in diabetic cardiomyopathy.

In GK rat ventricular myocytes with prolonged time to peak shortening, alterations in the LTCC current have not been replicated, although a faster decay of the calcium transient was observed [56]. Investigators have found no significant difference in the *Cacna1c* and *Cacna1g* gene expression or in the *Atp2a2* and *Calm1* genes, which have been described in the ZDF rats. However, dietary intake appears to have an impact on this model of rat. The impact of a sucrose-enriched diet to act as a model for poor dietary intake was assessed by feeding sucrose-enriched water to GK rats (GK-sucrose group) and compared to control GK rats (GK group) as well as control non-diabetic Wistar rats (Control group) and control non-diabetic Wistar rats who were also fed sucrose-enriched water (Control-sucrose group) [57]. The GK-sucrose group had reduced amplitudes of calcium transients compared to the GK group, as also seen in the control-sucrose group compared to the control group. The GK group showed a prolonged time to peak calcium transient compared to the control group. Gene expression analysis showed the GK myocytes had upregulation of the *Cacna1c*, *Cacna1g* and *Cacnb1* (LTCC beta 1 subunit) genes.

The disparity in findings seen by Salem et al. [56] compared to Gaber et al. [57] may be accounted for by the use of sucrose as a dietary modification. Furthermore, the former study was undertaken with rats aged 8–10 weeks compared to the much older age group that Gaber et al. studied given the 8-month dietary intervention. This suggests there may be changes in myocardial calcium handling and gene expression as a result of dietary intervention and aging.

#### 4.1.2. RyR2

Beyond the LTCC changes mentioned so far, there is further evidence of dysfunction at the level of the RyR2 [52]. A metabolic syndrome model of dogs was produced with the use of high-fat diet for six weeks. Although these dogs were normoglycemic, they did develop insulin resistance and had impaired cardiac index during exercise. The LV and right ventricular myocytes from these dogs were shown to have increased RyR2 phosphorylation at Ser2909 and RyR2 had a reduced ability to bind to [^3^H]ryanodine. This work was limited by the lack of quantification of the binding of calcium to the RyR2 receptor but there did appear to be a significant alteration in the binding affinity of RyR2, which may have resulted in abnormal calcium release from the SR and suggests abnormal myocardial calcium handling is present even in pre-diabetes.

#### 4.1.3. SERCA-2

Abnormalities and reduced expression of SERCA-2 have been demonstrated in diabetic mice with both impaired fractional shortening and diastolic dysfunction [54] and downregulation of genes encoding for SERCA-2 in the ZDF rat [55]. These findings are also seen in pre-diabetic rats, suggesting the changes may happen early before the onset of T2D [51]. This was demonstrated by feeding a high-sucrose diet to male Wistar rats for 9–12 weeks, producing an insulin resistant model with impaired cardiomyocyte shortening and relengthening, and comparing them to a control Wistar group who were fed a high-starch diet. A reduction in the rate of calcium removal from the cytosol in the insulin resistant group was found. The group further showed that this was related to a slower SERCA-2 uptake of calcium whereas the function of the sodium/calcium exchanger was normal.

In summary, dysregulation of calcium handling within cardiac myocytes across a range of diabetic and pre-diabetic animal models have been shown, with evidence of alterations at the level of the LTCC, RyR2 and SERCA-2 channels, albeit with some inconsistencies (Figure 3). None of these studies have reversed the diabetic phenotype and shown associated improvement of impaired myocardial calcium handling, which would be a crucial step towards determining causality. As with most animal models, these models have differences in physiological properties and gene expression compared to humans and they lack the heterogeneity seen in humans with T2D. Further work assessing calcium handling in humans is essential.

### 4.2. Human Studies

In vivo assessment of myocardial calcium handling in humans has been challenging, owing to the requirement of cardiac biopsy [58]. An alternative is the use of induced pluripotent stem cells (iPSCs). Nevertheless, very few cellular studies have explored dysregulated calcium handling in humans (Table 2).

In 2017, patients with severe aortic stenosis with preserved LV systolic function awaiting aortic valve replacement were categorized into T2D (*n* = 7) and non-T2D (*n* = 9) and had LV apical biopsies performed [59]. Although the aim of the study was to measure gene expression of channels associated with arrhythmias, quantitative polymerase chain reaction showed that patients with T2D had increased expression of *SLC8A1* encoding the sodium/calcium exchanger. This does not provide specific evidence for calcium dysregulation in T2D, but the increased expression of this gene may suggest changes in calcium efflux from the cardiomyocyte.

Further work has involved the use of iPSCs, which are somatic cells which have been reprogrammed into pluripotent stem cells by differential expression of a combination of transcription factors [60]. The advantage of such cells is they can be of human origin and also patient-specific. iPSCs have successfully been generated as models for other cardiac diseases [61,62]. Use has been limited in T2D due to the multifactorial complexity of T2D compared to monogenic disorders [63].

In 2018, Drawnel et al. [64] produced iPSC cardiac myocytes (iPSC-CM) which develop diabetic cardiomyopathy by exposure to a diabetic environment of glucose, endothelin-1 and cortisol. These cells demonstrated reduced calcium transient frequency and amplitude compared to iPSC-CM which weren’t exposed to a diabetic environment. Generation of iPSC lines from urine epithelial cells of patients with T2D and either urine or skin cells from healthy controls have also been performed [65]. These cells were then differentiated into iPSC-CM, and diabetic iPSC-CMs demonstrated several features of abnormal calcium handling compared to the iPSC-CMs from the healthy volunteers. These cells, however, do not truly recreate the multiple environmental, genetic and cellular factors present in the typical patient with T2D and extrapolation of findings into the clinical setting may not be appropriate [66].

**Table 2:** A summary of key studies on human specimens investigating myocardial calcium handling in diabetes.

Author, Year	Model	Method	Key Findings
Ashrafi et al., 2017 [59]	Human T2D vs. non-TDM	T2D (*n* = 7) and non-T2D (*n* = 9) with severe aortic stenosis patients undergoing valve replacement. LV apical biopsy taken, quantitative PCR for genetic analysis.	Increase expression of gene encoding sodium/calcium
Drawnel et al., 2018 [64]	iPSC-CMs in diabetic environment vs. normal environment	Produced iPSC-CMs and placed in diabetic environment	Reduced calcium transient frequency and amplitude
Tang et al., 2020 [65]	iPSCs from T2D vs. HV 2 patients (T2D) vs. 5 controls (HV)	T2D (*n* = 2) and HV (*n* = 5). iPSCs generated from urine or skin cells	Reduced calcium transient amplitude, shorter transient duration, shorter decay, slower maximal rising rate, slower maximal decay rate.

Abbreviations: T2D = type 2 diabetes, LV = left ventricle, PCR = polymerase chain reaction, iPSC = induced pluripotent stem cell, CM = cardiac myocyte, HV = healthy volunteer.

### 4.3. Mechanisms for Calcium Dysregulation in T2D

Mechanistic work characterizing changes in myocardial calcium handling in T2D continue to be explored in pre-clinical models. For example, diabetes leads to the accumulation of advanced glycation end products (AGE) which contribute to both micro- and macrovascular complications [67]. Pre-clinical models have shown a dose-dependent reduction of calcium transients when cardiac myocytes are treated with AGEs and this was associated with increased reactive oxygen species [68]. Furthermore, formation of AGEs on RyR2 results in impaired RyR2 function in diabetic rat models [69] as well as decreased SERCA-2 activity [70]. Interaction between AGEs and calcium handling may represent another link driving subsequent cardiac dysfunction and heart failure development.

Overall, experiments in end-stage heart failure and in models of T2D have demonstrated a common theme of abnormal calcium handling but with some important differences. In end-stage heart failure, a key pathological consequence appears to be cytosolic calcium overload which is a result of several changes including increased uptake by the LTCC, leakage at the RyR2 and abnormalities at the SERCA-2. In T2D, although some evidence for these changes do exist, key features appear to include reduced calcium transients and reduced expression of LTCC. Like all studies, each has limitations and there exists a significant challenge in obtaining and assessing myocardial tissue from patients with T2D. This has left a need for a non-invasive in vivo method of assessment of myocardial calcium handling.

## 5. Potential for Magnetic Resonance Imaging to Study In Vivo Myocardial Calcium Handling

### 5.1. Cardiovascular Magnetic Resonance

Cardiovascular magnetic resonance imaging is an advanced cross-sectional imaging technique that has become a key method of assessing several cardiovascular diseases [71]. Strong magnetic fields cause protons to align and tilt and following the generation and then removal of a radiofrequency pulse, protons undergo relaxation back to their net magnetization field which releases a radiofrequency signal. Relaxation of the protons can be defined by T1 and T2 relaxation times and different tissues within the body have different relaxation times which allowing differentiation [71,72]. For several decades gadolinium-based contrast agents have been used in MRI. These agents are highly paramagnetic and work by causing an increase in longitudinal relaxation rates and thus shortening T1 times [73,74,75,76]. Importantly, due to their large size, they cannot cross intact cell membranes and remain within the extracellular compartment and cannot provide further information of intracellular function [71].

### 5.2. Manganese and Cellular Function

In 1970, Ochi [77] investigated guinea-pig cardiac myocytes and measured membrane potentials with intracellular microelectrodes. When manganese ions were present in calcium- and sodium-free solutions, there was an increase in slow inward currents during depolarization. In 1976 [78], he went on to show that this inward current was not present if the LTCCs were blocked by lanthanum ions, suggesting that manganese ions are taken up by the LTCC. Further supporting evidence of this is that the LTCC blocker verapamil causes inhibition of manganese uptake, and a decrease in the concentration of calcium in the perfusate results in a significant increase in the uptake of manganese [79]. Manganese appears to remain within the intracellular space for longer in comparison to calcium. Once manganese enters the cell, it is taken up by the SR and also enters the mitochondria via the calcium uniporter, where it becomes bound to macromolecules. The remaining manganese within the cytosol binds to ATP [80]. These properties of manganese represent an opportunity for its utilization in the study of calcium handling.

### 5.3. Manganese-Enhanced MRI (MEMRI)

Manganese was the first contrast agent investigated for use in MRI due to its paramagnetic properties [81] and the previously licensed manganese contrast agent (Teslascan, GE healthcare AS) voluntarily withdrew from the market in 2012 for commercial reasons [82]. Initial challenges in the development of manganese as a contrast agent related to cardiotoxicity [83,84,85] and parkinsonism [81]. In order to overcome toxicity, two main methods were developed: chelation and the addition of chloride ions, of which chelation of manganese with dipyridoxyl diphosphate has been particularly successful. The use of manganese dipyridoxyl diphosphate allows a slower release and a more buffered concentration of manganese that is then available for uptake by the myocardium and other tissues [80]. Manganese can act as an analogue for calcium uptake making it an attractive contrast agent for investigation of in vivo myocardial calcium handling.

To test if manganese can assess myocardial calcium uptake, Hu et al. [86] used manganese chloride as a contrast agent with concurrent use of either dobutamine or diltiazem. Dobutamine is a positive inotrope due to increased calcium influx and diltiazem is a negative inotrope with reduced calcium influx. Myocardial signal intensity was enhanced on T1-weighted imaging with the use of manganese chloride without any significant impact on cardiac function. Importantly, they showed increased signal enhancement and rates of enhancement with dobutamine and decreased signal enhancement and rates of enhancement with diltiazem, suggesting that manganese can be used as a method of assessing myocardial calcium influx.

### 5.4. Clinical Studies Using MEMRI

In 2004, Skjold et al. [87] demonstrated the use of MEMRI in healthy volunteers using manganese dipyridoxyl diphosphate. They found that participants who were given 5 µmol/kg had a significant and prolonged rise in myocardial relaxation rates, but this rise did not increase linearly with increasing doses beyond 10 µmol/kg. This group then went on to describe an in vivo unidirectional manganese influx constant (Ki), calculated using a two-compartment Patlak model, which could be used to assess myocardial calcium uptake [88]. MEMRI has been investigated as an agent for the assessment of cardiac pathology (Table 3).

### 5.5. MEMRI to Study Myocardial Infarction

There has been some interest in using MEMRI in patients with myocardial infarction as a means of assessing viability. In patients with recent infarction, MEMRI was shown to have higher T1 relaxation rates in non-infarcted areas compared to infarcted areas [89]. Spath et al. [91] also evaluated patients with recent infarction (*n* = 20) and compared MEMRI scans with healthy volunteers (*n* = 20) to demonstrate Ki values differed between infarcted, peri-infarct and remote myocardium in a stepwise fashion (11.7 ± 3.5 vs. 16.7 ± 3.8 vs. 23.0 ± 3.0 mL/100 g/min). The reason for such changes is thought to be due to a reduced uptake of manganese in infarcted myocardium resulting in the lack of change in T1 times.

### 5.6. MEMRI in Cardiomyopathies

MEMRI has been used to investigate patients with non-ischemic dilated and hypertrophic cardiomyopathy [90]. Compared to healthy volunteers (*n* = 20), patients with dilated cardiomyopathy (*n* = 10) had a lower rate of myocardial manganese uptake (Ki 23 ± 4 vs. 19 ± 4 mL/100 g of tissue/min, *p* = 0.0068). In patients with hypertrophic cardiomyopathy (*n* = 17) there were lower Ki values in those with fibrosis compared to without fibrosis, who further had lower Ki values compared to healthy volunteers (13 ± 4 vs. 19 ± 3 vs. 23 ± 4 mL/100 g of tissue/min, *p* < 0.001). In patients with Takotsubo cardiomyopathy, manganese uptake is also reduced compared to controls (5.1 ± 0.5 vs. 8.2 ± 1.1 mL/100 g of tissue/min, *p* < 0.001) and this persisted despite restoration of normal LV systolic function [92].

### 5.7. MEMRI in Diabetes

**Figure 4:**
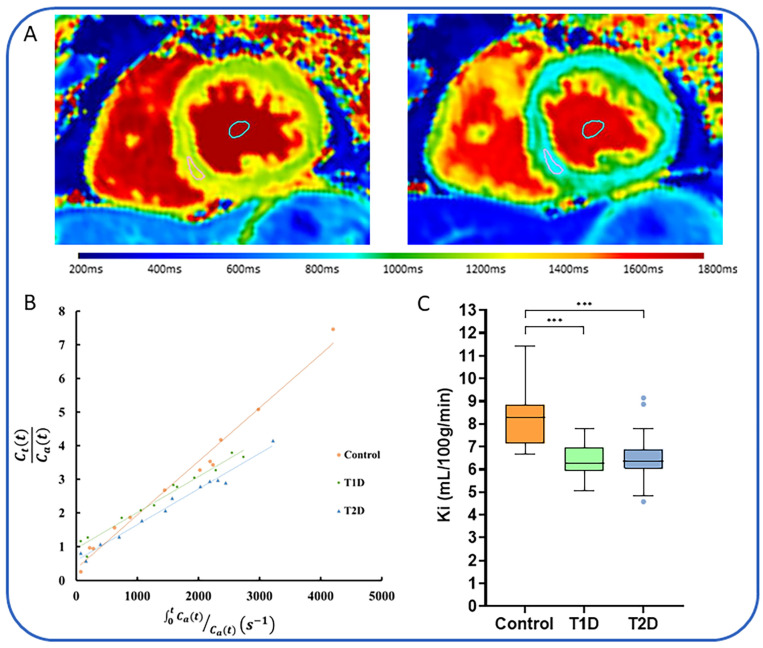
Manganese-enhanced magnetic resonance imaging. Serial T1 mapping using manganese-enhanced MRI can be used to assess calcium uptake in-vivo (**A**). T1 values can be entered into a Patlak model (**B**) to produce a manganese influx constant (Ki). Patients with T1D and T2D have reduced Ki compared to Controls (**C**). Abbreviations: T1D = type 1 diabetes, T2D = type 2 diabetes, Ki = manganese influx constant. Scale represents T1 times. Figure adapted with permission from Dattani et al. [93], Copyright 2023, Elsevier.

Limited in vivo work has so far been performed using MEMRI in people with T2D. Our group has recently published the first study assessing in vivo myocardial calcium uptake using MEMRI in people with type 1 diabetes and T2D (Figure 4) [93]. Using a further optimized kinetic model, we showed that people with both forms of diabetes had impaired myocardial calcium uptake in comparison to age-matched healthy volunteers (6.43 ± 0.77 vs. 6.47 ± 0.99 vs. 8.29 ± 1.31 mL/100 g of tissue/min, *p* < 0.001). In this study, the participants had no prevalent cardiac disease and no apparent LV systolic or diastolic dysfunction, suggesting that abnormal calcium uptake may be an early pathological feature of diabetic cardiomyopathy. Longitudinal studies with serial follow-up imaging are required to evaluate the association between reduced calcium uptake and downstream development of HF.

## 6. Calcium Handling as a Therapeutic Target in Diabetes

Numerous advances have been made in the pharmacological management of HF including angiotensin receptor/neprilysin inhibitors and sodium glucose co-transporter 2 (SGLT-2) inhibitors [94]. Although these contemporary medications have demonstrated reduced HF outcomes, they have not cured the underlying disease as evidenced by the EMPEROR-Reduced trial [95] in which 22% of patients with T2D still reached primary endpoint of cardiovascular death or hospitalization for HF despite being on recommended therapy in addition to an SGLT-2 inhibitor. Moreover, limited pharmacological therapies are available for patients with HF with preserved ejection fraction [94] which commonly affects those with T2D. Identification of specific biological pathways driving progression of diabetic cardiomyopathy may unveil important targets for prevention and treatment [96], and this has been highlighted as an urgent unmet need by major international cardiology and diabetes societies [97,98]. The unravelling of perturbations in calcium handling in T2D may allow the use of precision medicine in T2D-related HF and several agents could be used to reverse the dysregulated myocardial calcium seen (Figure 1).

### 6.1. Glucagon-like Peptide-1 (GLP-1) Receptor Agonists

Various GLP-1 receptor agonists have already been developed for the treatment of T2D but GLP-1 receptors are present in a multitude of organs including the heart. When mouse atrial myocytes are treated with GLP-1, there is an increase in calcium transients and SR calcium contents as well as decreased phosphorylation of RyR2 [99]. These findings have been corroborated in adult canine cardiomyocytes, where extracellular perfusion of GLP-1 results in a 23% increase in LTCC currents, but the pre-administration of a GLP-1 receptor antagonist abolishes this increase [100]. Moreover, intracellular dialysis with a protein kinase A inhibitor prevents this increase in LTCC current. Overall, these results suggests that GLP-1 enhances the LTCC current via the cyclic adenosine monophosphate-dependent protein kinase A pathway. This could indicate a future role of GLP-1 receptor agonists in diabetic cardiomyopathy but the impact of these drugs on HF requires more evidence [101]. An ongoing randomized placebo-controlled trial will assess the impact of semaglutide in those with HF with preserved ejection fraction, obesity and T2D [102].

### 6.2. SGLT-2 Inhibitors

SGLT-1 receptors are present on the cardiomyocyte sarcolemma and recent work has shown the existence of SGLT-2 in human cardiomyocytes [103]. Increased expression of these receptors and sodium-hydrogen exchanger-1 are seen in T2D and HF [104] resulting in increased intracellular sodium concentration. This causes increased influx at the sodium-calcium exchanger at the sarcolemma and efflux from the mitochondria, leading to intracellular calcium overload which results in impaired calcium transients and reduced SR calcium stores [105].

SGLT-2 inhibitors have now been ingrained into international guidelines for the management of HF [94] given their significant benefit on outcomes, but their mechanism of action still remains to be fully elucidated. Amongst potential mechanisms lies their impact on myocardial calcium handling. Cardiomyocytes from streptozotocin-induced diabetic rats when treated with empagliflozin have greater LTCC and intracellular calcium currents, and lower sodium-hydrogen exchanger-1 currents [106]. Exposure of mouse cardiomyocytes to empagliflozin, dapagliflozin and canagliflozin, has also shown inhibition of the sodium-hydrogen exchanger-1 [107] suggesting a class effect. It is thought that the non-specific inhibitory actions of SGLT-2 inhibitors on SGLT-1 and the sodium-hydrogen exchanger-1 may lead to reversal of intracellular calcium overload.

### 6.3. Calcium Sensitizers

Levosimendan is a calcium sensitizer which binds to the N-terminus of troponin C causing stabilization of the calcium-troponin C complex, accelerating cross-bridge formation and preventing dissociation [108]. There does appear to be a wider impact of the drug though. For example, in cardiac slices from wistar rats with streptozotocin-induced diabetic cardiomyopathy, levosimendan increases the expression of SERCA-2 and sodium-calcium exchanger-1 [109].

### 6.4. Protein Kinases

Protein kinase inhibitors have revolutionized cancer therapeutics [110]. The expression of protein kinase C-βII is increased in human HF [111]. Cardiomyocytes from a mouse model of HF with over-expression of protein kinase C-βII showed reduction in the shortening and rate of re-lengthening of the cardiomyocytes but no difference in calcium signals suggesting decreased myofilament response to calcium. Importantly, the presence of a protein kinase C-βII inhibitor improved cardiomyocyte function [112]. Protein kinase C also leads to decreased activity of SERCA-2 and causes dephosphorylation of phospholambin. Thus, protein kinase C-β inhibitors, such as LY-379196, may aide in cardiac calcium dysregulation and improve calcium sensitization [108].

Furthermore, calmodulin-dependent protein kinase-II may be an important future therapeutic target [113] as it phosphorylates the LTCC which allows an increase in calcium influx. It also facilitates the phosphorylation of RyR2 to increase the release of calcium and catalyzes the phosphorylation of phospholambin which leads to a reduction of phopholambin’s inhibitory activity on SERCA-2.

### 6.5. Other Drug Therapy

Istaroxime is a steroidal drug which has beneficial effects on calcium handling [114]. In streptozotocin-treated diabetic rats with diastolic dysfunction, istaroxime infusion was able to improve diastolic dysfunction by stimulating SERCA-2 [115].

Ranolazine is a commonly used anti-anginal medication. In a diabetic rat model, the use of ranolazine attenuated the cardiac impact of hyperglycemia and may help prevent the occurrence of diabetic cardiomyopathy [116]. Whilst this was attributed to the activation of the NOTCH1/NRG1 pathway which is related to apoptosis, ranolazine has wider effects on calcium handling by enhancing the sodium-calcium exchanger but these were not assessed during this study and further work is required.

### 6.6. Lifestyle Modification

Beyond pharmacological therapies, exercise and diet have shown important cardiovascular benefits in T2D [18]. Using a mouse model of T2D, 13 weeks of aerobic interval training almost completely resolved contractile dysfunction and demonstrated significant improvements in calcium handling including calcium amplitudes, and SERCA-2 and sodium-calcium exchanger activity [54].

None of these therapeutic targets have been tested in vivo to assess their impact on myocardial calcium handling. The availability of MEMRI, however, potentially allows this. Indeed, MEMRI is currently being used in a trial to assess the impact of an SGLT-2 inhibitor on calcium handling in patients with symptomatic HF with and without T2D (NCT04591639) and a randomized controlled trial will assess the role of diet and exercise on calcium handling in obese patients with T2D [117].

## 7. Conclusions

Mounting evidence from animal models suggests that myocardial calcium handling is impaired in T2D and this may play a key role in the development of diabetic cardiomyopathy. We have recently demonstrated this to be true in people with T2D in vivo using MEMRI, which may open the door for the technique’s use as a future surrogate marker for monitoring the response to novel therapeutic agents, with the end-goal of preventing and treating diabetic cardiomyopathy.

## Figures and Tables

**Table 1:** A summary of key experimental studies investigating myocardial calcium handling in animal models of diabetes.

Author, Year	Model	Method	Key Findings
Wold et al., 2005 [51]	High sucrose diet rats (insulin resistant model) vs. controls	9–12 weeks dietary intervention of high sucrose diet. Fluo-3, immunoblot assays	Insulin resistant rats have slower cytosolic calcium removal and slower SERCA2 calcium uptake.
Dincer et al., 2006 [52]	High-fat diet (metabolic syndrome model) vs. normal diet in dogs (control).	Six-week dietary intervention. RNA samples from RA, RV and LV, reverse transcription and RT-PCR. Western blot analyses. Ser^2809^ phospho-specific antibodies and [^3^H]ryanodine for RyR2 assessment.	Increased RyR2 phosphorylation at Ser^2809^ and reduced ability of the RyR2 bind to [^3^H]ryanodine. No change in the gene expression of RyR2.
Pereira et al., 2006 [53]	Diabetic mouse (db/db) vs. control mouse (+/+).	Two-photon microscopy, confocal microscopy, Fluo-3, whole-cell patch clamp technique, western blots	Decreased intracellular calcium transients, LTCC expression, calcium current, SR calcium load, ryanodine receptors, and increased calcium efflux via sodium/calcium exchanger
Stølen et al., 2009 [54]	Diabetic mouse (db/db) vs. heterozygote controls	Fura-2 and Fluo-3 indicators, bipolar electric pulses, western blots, RT-PCR, exercise training, echocardiography	Sedentary db/db mice had lower calcium release, lower SR calcium load, slower calcium decay, reduced T tubule density, reduced expression of SERCA-2a. All features improved with exercise training.
Howarth et al., 2011 [55]	ZDF rat vs. age-matched control lean	Fura-2, whole cell patch clamp techniques, RNA sampling from LV apex with reverse transcription and gene expression assays.	Upregulation of genes for LTCC subunits. Downregulation of genes for SERCA2 and Calmodulin 1. Reduced LTCC current in ZDF rats, prolonged inactivation of LTCC current and prolonged time to peak calcium transient. No change in SR release, uptake and calcium content.
Salem et al., 2012 [56]	GK rats vs. Wistar control rats	Fura-2, RNA sampling from LV apex with reverse transcription and gene expression assays	No change in LTCC subunit gene expression. Upregulation of TTCC, potassium and sodium channel gene expression. No change in intracellular calcium amplitude but faster decay of calcium transient seen.
Gaber et al., 2014 [57]	GK vs. GK-sucrose vs. Control (Wistar) vs. Control-sucrose rats	Eight-month intervention of sucrose-enriched water. Fura-2 used. Ventricular RNA samples with reverse transcription and gene expression assays	Prolonged time to peak shortening of myocyte in GK group, reduced amplitude of myocyte shortening and reduced amplitude of calcium transients in the sucrose groups. Upregulation of genes for LTCC subunits in GK rats and RyR2 in control-sucrose rats.

Abbreviations: LTCC = voltage gated L-type calcium channel, SR = sarcoplasmic reticulum, RT-PCR = reverse transcription polymerase chain reaction, SERCA = sarcoplasmic reticulum calcium ATPase, ZDF = Zucker diabetic fatty, RNA = ribonucleic acid, LV = left ventricle, GK = Goto-Kakizaki, TTCC = voltage gated T-type calcium channel, RyR2 = ryanodine receptor 2, RA = right atrium, RV = right ventricle.

**Table 3:** Key clinical studies assessing the use of manganese-enhanced MRI in cardiac pathology.

Author, Year	Participants	Inclusion Criteria	Exclusion Criteria	Method	Key Findings
Skjold et al., 2007 [89]	10	Males (*n* = 8) and females, age 37–75, MI within 12 weeks with PCI.		Given 5 µmol/kg of MnDPDP over 5 min, 1.5T scanner used to obtain images pre- and post-contrast.	Higher T1 relaxation rates in non-infarcted myocardium vs. infarcted myocardium.
Spath et al., 2020 [90]	47	HVs (*n* = 20), non-ischaemic DCM (*n* = 10) and HCM (*n* = 17)	NYHA class IV, contraindications to MRI or MnDPDP.	Open-label, observational cohort study. Gadolinium-enhanced MRI and MEMRI >48 h apart. T1 mapping performed using 3T scanner every 2.5 min. MnDPDP administered at dose of 5 µmol/kg at rate of 1 mL/min.	Patients had lower mean reductions in T1 values and lower Ki following MnDPDP compared to HVs.
Spath et al., 2021 [91]	40	HV (*n* = 20). STEMI patients (*n* = 20) with proven LMS, LAD or multi-vessel disease, clinically stable, EF < 50%,	NYHA class IV, contraindications to MRI or MnDPDP.	Open-label, observational cohort study. Gadolinium-enhanced MRI and MEMRI >48 h apart, within 7 days of revascularisation and at 3 months. T1 mapping performed using 3T scanner every 2.5 min. MnDPDP administered at dose of 5 µmol/kg at rate of 1 mL/min.	Infarcted myocardium had higher T1 values at 40 min compared to remote myocardium and HVs. Ki values were lower in infarcted compared to peri-infarct myocardium, which were also lower than remote myocardium.
Singh et al., 2022 [92]	40	HV (*n* = 20) and Takotsubo cardiomyopathy patients (*n* = 20)		Prospective, case-control study using MEMRI. T1 mapping performed using 3T scanner every 2.5 min. MnDPDP administered at dose of 5 µmol/kg at rate of 1 mL/min.	Patients with Takotsubo had lower mean Ki which persisted despite recovery of LV systolic function.
Dattani et al., 2023 [93]	60	HV (*n* = 11), T1D (*n* = 19), T2D (*n* = 30).	Symptoms or history of cardiac disease	Prospective, case-control study using MEMRI. T1 mapping performed using 3T scanner every 2.5 min. MnDPDP administered at dose of 5 µmol/kg at rate of 1 mL/min.	Patients with T1D and T2D had lower mean Ki compared to controls.

Abbreviations: HV = healthy volunteer, MnDPDP = manganese dipyridoxyl diphosphate, MI = myocardial infarction, PCI = percutaneous coronary intervention, DCM = dilated cardiomyopathy, HCM = hypertrophic cardiomyopathy, NYHA = New York Heart Association, eGFR = estimated glomerular filtration rate, STEMI = ST-elevation myocardial infarction, LMS = left main stem, LAD = left anterior descending, Ki = manganese influx constant.
